# Age-Stratified Serum Biomarkers for Discriminating Clinically Defined Cognitive Impairment in Neurological Inpatients: A Cross-Sectional Study

**DOI:** 10.3390/brainsci16101080

**Published:** 2026-10-08

**Authors:** Dan Gu, Yu Lei, Jiayi Lu, Wenmin Yi, Qinbin Zhang, Yuxuan He, Chuanling Wang, Zhiyou Cai

**Affiliations:** 1 Department of Neurology, People’s Hospital of Chongqing Liangjiang New Area, Liangjiang Hospital of Chongqing Medical University, No. 2, Jinkai Avenue, Liangjiang New Area, Chongqing 401121, China; 15002356253@163.com (D.G.); 19722761629@163.com (Y.L.); 17570838930@163.com (J.L.); yiwenmin612@163.com (W.Y.); 19942334440@163.com (Q.Z.); hyx0048@126.com (Y.H.); 2 Department of Clinical Medicine, Chongqing Medical University, NO.1 Yixueyuan Road, Yuzhong District, Chongqing 400016, China; 3 Chongqing Key Laboratory of Neurodegenerative Diseases, No. 118 Xingguang Avenue, Liangjiang New Area, Chongqing 401147, China; 4 Department of Pathophysiology, Chongqing Medical University, NO.1 Yixueyuan Road, Yuzhong District, Chongqing 400016, China

**Keywords:** age, cognitive impairment, tau protein, neurofilament light chain, glial fibrillary acidic protein

## Abstract

**Highlights:**

**What are the main findings?**

- Age-stratified analysis revealed differential biomarker performance patterns in cognitive impairment: p-Tau181 showed relatively better discriminative performance in patients aged <65 years (AUC = 0.6212), while GFAP (AUC = 0.7776) and NfL (AUC = 0.7329) showed numerically higher AUC values in patients aged ≥65 years.
- GFAP showed no significant correlation with age after adjusting for cognitive status (r = 0.062, *p* = 0.207), suggesting that its elevation may primarily reflect cognitive impairment burden rather than physiological aging.

**What are the implications of the main findings?**

- The differential performance of biomarkers across age groups suggests that age-specific interpretation may be important when using serum biomarkers for cognitive assessment in neurological inpatient settings.
- The modest discriminative performance of individual biomarkers underscores the need for comprehensive clinical evaluation rather than sole reliance on any single serum biomarker, and highlights the necessity for future studies with formal age-by-biomarker interaction testing and multi-marker approaches.

**Abstract:**

**Background:** With the aging of the global population and the increasing prevalence of cognitive dysfunction worldwide, early identification of cognitive impairment has become an important public health issue. This study aims to investigate the correlation between peripheral serum neurodegenerative biomarkers and age, as well as the discriminative efficacy of cognitive impairment across different age cohorts. Notably, subjects were grouped by clinical cognitive assessment without pathological confirmation via amyloid/tau PET or cerebrospinal fluid testing to define Alzheimer’s disease pathology. **Methods:** A cross-sectional study enrolled 425 inpatients from the neurology department. Nonparametric tests were used to explore the correlation between age and peripheral serum biomarkers, as well as intergroup differences in biomarker levels among different cognitive statuses. Age-stratified logistic regression models were constructed to evaluate the association between biomarkers and cognitive impairment, with odds ratios (OR) and 95% confidence intervals (CI) calculated. Receiver operating characteristic (ROC) curves and corresponding area under the curve (AUC) were adopted to assess the discriminative performance of each indicator. In this study, cognitive impairment was defined clinically based on MoCA and clinical assessment, not biomarker-confirmed Alzheimer’s disease pathology. **Results:** Except for glial fibrillary acidic protein (GFAP) (r = 0.062, *p* = 0.207), all detected biomarkers were significantly correlated with age. Among them, p-Tau181 (*r* = 0.288, *p* < 0.001), p-Tau217 (*r* = 0.275, *p* < 0.001), Aβ1-42 (*r* = 0.132, *p* = 0.007), Aβ1-40 (*r* = 0.254, *p* < 0.001), and NfL (*r* = 0.200, *p* < 0.001) were positively associated with age, while Aβ42/40 ratio was negatively correlated with age (*r* = −0.253, *p* < 0.001). After age-based grouping, phosphorylated tau protein (p-Tau)181 showed relatively better discriminative performance in the <65 years group (AUC = 0.6212, 95% *CI*: 0.537–0.705); GFAP (AUC = 0.7776, 95% *CI*: 0.718–0.837) and NfL (AUC = 0.7329, 95% *CI*: 0.670–0.796) showed higher AUC values among participants aged ≥65 years. **Conclusions:** The correlation and discriminative efficacy of peripheral serum biomarkers for clinically defined cognitive impairment showed differences across age groups in this exploratory analysis. Within this inpatient cohort, p-Tau181 showed relatively better discriminative performance in the <65 years group, while GFAP and NfL showed numerically higher AUC values in the ≥65 years group. These exploratory findings suggest age-related differences in biomarker performance patterns that warrant further investigation in independent cohorts. The modest AUC (0.6212) for p-Tau181 in the younger group suggests that while it offers a statistical advantage, its clinical utility as a standalone screening tool remains limited, reinforcing the need for comprehensive clinical evaluation. Our results provide exploratory evidence for age-stratified minimally invasive serum-based assessment of clinically defined cognitive impairment in neurological inpatients, and cannot be directly generalized to community populations or used for definite Alzheimer’s disease pathological screening; rather, our results serve as a reference for age-stratified screening in clinical inpatient settings rather than standalone diagnostic criteria.

## 1. Introduction

With the acceleration of global population ageing, cognitive impairments and multiple subtypes of dementia have become an increasingly severe public health challenge, among which Alzheimer’s disease (AD) is one of the most common causes. Statistics indicate that the number of individuals affected by dementia continues to rise globally, imposing a substantial social and economic burden [1]. In China, where the pace of ageing significantly exceeds the global average, the prevalence of cognitive impairment and dementia is particularly pronounced, necessitating urgent and effective countermeasures [2,3]. The core pathological feature of Alzheimer’s disease is progressive neuronal degeneration. The clinical course typically commences with subjective cognitive decline (SCD), progresses through mild cognitive impairment (MCI)—a critical early symptomatic stage—and ultimately advances to varying degrees of dementia, encompassing both moderate dementia and severe dementia [4,5]. Accurate identification of the MCI stage is crucial for delaying or halting progression to dementia. However, clinical practice currently faces challenges in recognising and differentiating cognitive impairment, particularly early-stage MCI [6]. Currently, the gold standard for confirming AD-specific pathological changes relies on detecting specific biomarkers such as Amyloid-beta42 (Aβ42), Amyloid-beta40 (Aβ40), Phosphorylated tau protein (p-Tau), and total tau protein (t-Tau) in cerebrospinal fluid (CSF), as well as amyloid positron emission tomography (PET) and tau-PET imaging techniques [7,8]. These methods directly reflect core AD pathophysiological changes, such as β-amyloid deposition and hyperphosphorylated tau forming neurofibrillary tangles in the brain, offering high diagnostic accuracy for definitive AD diagnosis [9]. However, in clinical practice, lumbar puncture for CSF collection is an invasive procedure carrying risks of complications like infection and bleeding. This results in generally low patient acceptance and requires high technical proficiency from physicians [10,11,12]. PET scans, meanwhile, face challenges including extremely high costs, risks of radiation exposure, and low equipment availability, making widespread adoption equally difficult [13,14]. These limitations prevent existing diagnostic tools from meeting the urgent need for early, widespread screening and dynamic monitoring of cognitive impairment in an aging society, severely restricting their feasibility for large-scale population screening and routine clinical application [15].

It is well established that the neuropathological hallmarks of Alzheimer’s disease, such as amyloid-beta deposition and tau hyperphosphorylation, begin to accumulate decades before the onset of overt clinical symptoms [16]. This protracted preclinical phase underscores the pressing need for minimally invasive, cost-effective biomarkers that can identify individuals at risk in the early, asymptomatic stages, enabling timely intervention.

Considering these challenges, developing a minimally invasive, convenient, cost-effective, and reliable tool for early cognitive assessment has become an urgent priority. Peripheral serum neurodegenerative biomarkers have emerged as a research hotspot in recent years, and landmark cohort studies have validated the discriminative potential of plasma p-tau, glial fibrillary acidic protein (GFAP), and neurofilament light chain (NfL) for cognitive impairment associated with Alzheimer’s disease pathology [17,18,19,20,21,22]. With the maturation and application of immunoassay technologies, stable detection of neurodegeneration-related pathological proteins, neuropathological damage, and inflammatory markers in blood has become feasible, providing a viable pathway for clinical translation [23,24,25]. Although existing evidence indicates that the levels of these serum biomarkers correlate with brain pathology and cognitive decline [24,26,27,28,29], there remains a lack of systematic evaluation of whether their discriminatory efficacy for cognitive impairment varies across age groups, especially since data from hospitalized neurological cohorts in Chinese populations are scarce. Notably, the present study enrolled neurological inpatients, and cognitive status was defined solely by comprehensive clinical cognitive scales without PET or CSF pathological testing; the findings are mainly applicable to inpatient populations rather than community-based subjects. Therefore, this paper proposes a systematic exploration of their discriminative value for clinically defined cognitive impairment through an age-stratified strategy, aiming to provide a reference for establishing an efficient, precise, and minimally invasive early screening pathway for cognitive impairment.

## 2. Methods

### 2.1. Research Subjects and Data Collection

This cross-sectional study prospectively enrolled 425 inpatients consecutively admitted to the neurology department of a tertiary hospital in southwestern China between October and December 2025. Inclusion criteria were: (1) age ≥18 years; (2) completion of a comprehensive clinical, cognitive, and psychological assessment; (3) venous blood sampling within 48 h of admission; (4) complete and accurate documentation. Exclusion criteria comprised: (1) confirmed diagnosis of non-AD-related dementia such as vascular dementia, Lewy body dementia, or frontotemporal dementia; (2) severe hepatic or renal impairment (eGFR < 30 mL/min/1.73 m^2^); (3) history of acute stroke, intracranial infection or tumour, or psychiatric disorder; (4) inability to cooperate in completing the questionnaire or ongoing treatment with sedatives, anticholinergics, or other psychotropic medications that could significantly affect cognitive performance. Notably, all participants were categorized into different cognitive groups based solely on clinical neuropsychological scales; no amyloid/tau-PET or CSF biomarker testing was performed to confirm Alzheimer‘s disease pathological changes.

This study complies with the Declaration of Helsinki. Approval has been obtained from the Ethics Review Committee of the People’s Hospital of Chongqing Liangjiang New Area (Approval Number: 2026003).

### 2.2. Cognitive and Psychological Assessment Methods

Cognitive function was assessed using the internationally recognized Montreal Cognitive Assessment (MoCA) [30]. The MoCA has a maximum score of 30 points, with grouping criteria referencing the Chinese Guidelines for the Diagnosis and Management of Dementia and Cognitive Impairment [31]. In this study, cognitive impairment was defined as a MoCA score <26 for participants with >6 years of education, and <24 for those with ≤6 years of education, consistent with Chinese normative data [32]. The assessment of anxiety and depressive states employs the Hamilton Anxiety Scale (HAMA) and the Hamilton Depression Scale (HAMD) [33,34]. All scale items underwent cultural adaptation to ensure semantic equivalence; for instance, descriptions within the ‘somatic anxiety’ section were localised to align with common presenting complaints among Chinese patients. Our psychological assessments were conducted under mutual masking: assessors had no access to patients’ blood test results, while laboratory personnel were unaware of psychological assessment data. All scales were completed within 72 h of admission by two certified neuropsychologists in quiet, independent consultation rooms to minimize interference. For individuals with lower educational attainment, standardized instructions supplemented with pictorial aids were employed to ensure assessment reliability. Inter-rater reliability was assessed using Intraclass Correlation Coefficients (ICCs). Fifty randomly selected scales were independently scored, yielding an ICC of 0.973 (95% *CI*: 0.952–0.984) for the HAMA and 0.982 (95% *CI*: 0.967–0.990) for the HAMD. Subjects were stratified into two age cohorts based on the commonly used clinical threshold: <65 years (18–64 years) and ≥65 years (65–94 years) for subsequent subgroup analyses.

### 2.3. Biomarker Detection

Quantitative detection of biomarkers was performed using a fully automated chemiluminescent immunoassay analyser (Shenzhen Yingkai Biotechnology Co., Ltd., Shenzhen, China, Shine i2910), with kits provided by Hunan Nuoweizan Medical Technology Co., Ltd., Chenzhou, China (catalog number: 250707A). Serum samples were stored at −80 °C for a median duration of 5 days and underwent one freeze–thaw cycle before analysis. All samples were analyzed in a single batch to minimize inter-assay variability. This technique offers distinct advantages in sensitivity and specificity and is widely employed in the analysis of serum biomarkers for neurodegenerative diseases [35,36,37]. Specific assay parameters included amyloid beta fragments (Aβ1-40, Aβ1-42, and Aβ42/40), GFAP, NfL, and phosphorylated tau protein (p-Tau181, p-Tau217). All blood sample processing strictly adhered to standardised operating procedures. Centrifugation (3500 rpm, 10 min, at room temperature) is performed within 2 h of collection, and the separated serum is immediately aliquoted for testing. Each batch of testing included manufacturer-provided quality control materials (high, medium, and low concentrations) and 10% random duplicate samples to monitor intra- and inter-batch precision. Quality control values were required to fall within ±20% of the target range, with duplicate samples exhibiting a coefficient of variation (CV%) below 15% [38]. Given that the assays were designed for serum samples, all measurements were performed according to the manufacturer’s instructions; however, independent analytical validation specifically for serum was not performed in our laboratory.

### 2.4. Statistical Analysis

The primary dependent variable was cognitive impairment status, which was dichotomized based on the MoCA-defined cognitive groups: participants classified as cognitively normal (CN) served as the reference group, while those classified as MCI, moderate dementia, or severe dementia were grouped into the cognitively impaired category. The independent variables of interest were the seven serum biomarkers: p-Tau181, p-Tau217, GFAP, NfL, Aβ1-40, Aβ1-42, and the Aβ42/40 ratio. Age was treated both as a continuous variable for correlation analyses and as a stratifying variable for subgroup analyses using a cutoff of 65 years. Confounding variables were prespecified based on clinical knowledge and data availability and included sex, anxiety status, and depression status. In the stratified analyses, age group (<65 years vs. ≥65 years) served as the stratifying variable.

Statistical analyses were performed using SPSS version 29.0 (IBM Corp., Armonk, NY, USA) and GraphPad Prism 11.0 (GraphPad Software, Boston, MA, USA). The normality of continuous variables was assessed using the Shapiro–Wilk test. As the data were not normally distributed, descriptive statistics are presented as median (Q1, Q3), where Q1 and Q3 denote the 25th and 75th percentiles, respectively. Categorical variables are presented as frequencies and percentages. For comparisons of baseline characteristics between age groups (<65 years vs. ≥65 years), the Mann–Whitney U test was used for continuous variables and the chi-square test for categorical variables. For comparisons of biomarker levels across multiple cognitive status groups (CN, MCI, moderate dementia, and severe dementia), the Kruskal–Wallis H test was employed. Where inter-group differences were statistically significant (*p* < 0.05), post-hoc pairwise comparisons were performed using the Dunn test with Bonferroni correction [39]. Spearman’s rank correlation coefficient was used to investigate bivariate associations between age and biomarkers, as the biomarker data were not normally distributed and the relationships were considered potentially monotonic rather than necessarily linear [40]. Partial Spearman correlation coefficients were calculated using the Partial Correlations procedure in SPSS, with sex and cognitive status entered as control variables to assess the independent association between age and each biomarker. Multivariable binary logistic regression models were constructed separately for the <65 years and ≥65 years age groups to evaluate the association between each biomarker and cognitive impairment status (impaired vs. normal). Each biomarker was entered into a separate logistic regression model. All models were adjusted for sex, anxiety status, and depression status to mitigate potential confounding. Odds ratios (ORs) and their 95% confidence intervals (CIs) were calculated. Receiver operating characteristic (ROC) curves were generated for each biomarker in each age group, and the area under the curve (AUC) was calculated to quantify discriminative performance. All statistical tests were two-tailed, with a significance level set at α = 0.05.

## 3. Results

### 3.1. Comparison of Baseline Characteristics and Age Stratification

**Figure 1:**
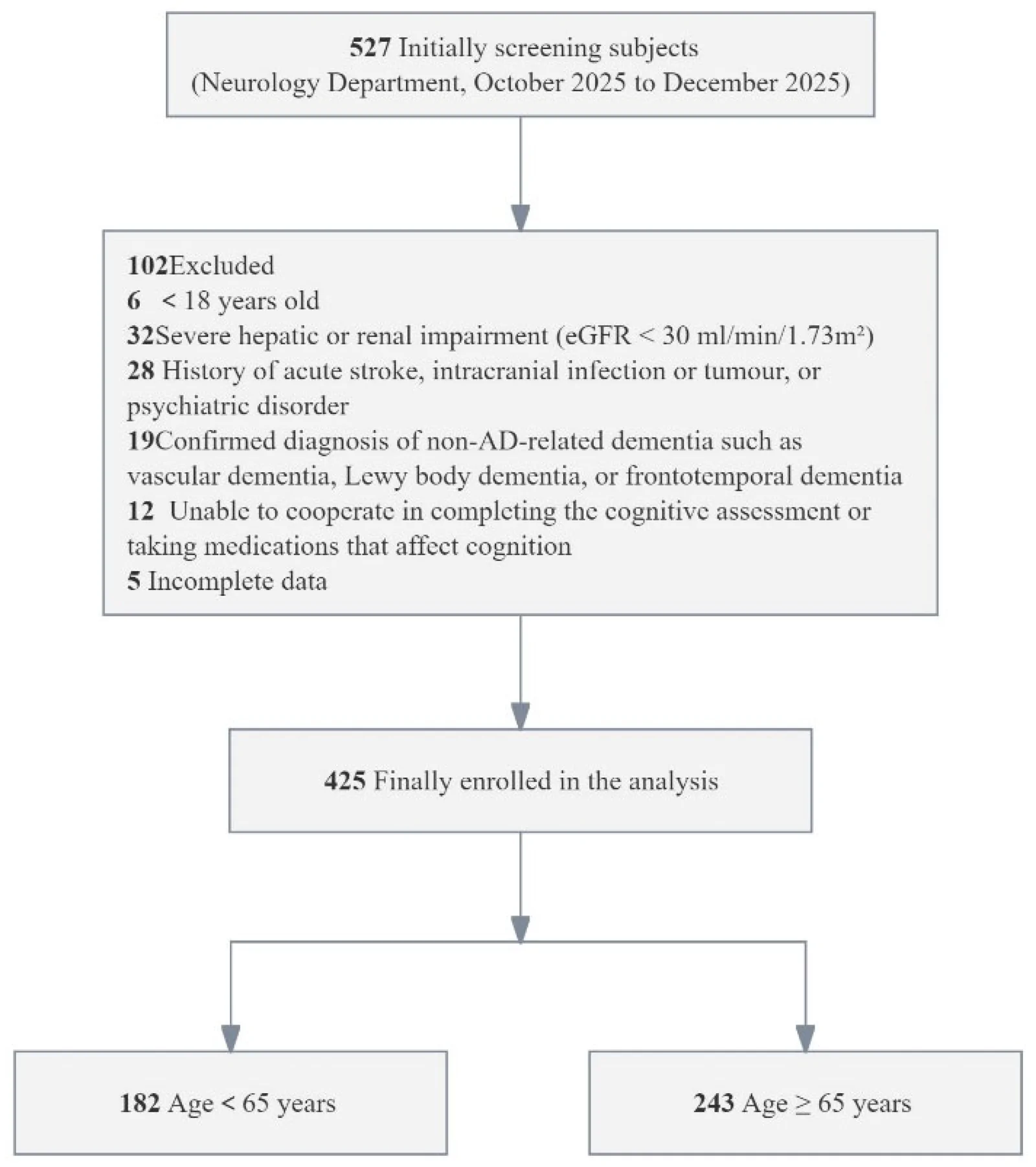
A participant flow diagram. Sample sizes at each stage are shown in the figure.

A total of 527 patients were initially screened during the study period. Of these, 102 were excluded. This study finally included 425 neurology inpatients, dividing the overall sample into a <65 years group (*n* = 182) and a ≥65 years group (*n* = 243) based on the commonly used clinical threshold of 65 years (Figure 1). Systematic comparisons were conducted between the two groups regarding demographic characteristics, cognitive function, and levels of serum-based neurodegeneration-associated biomarkers (Table 1).

Analysis of demographic characteristics revealed no statistically significant difference in sex distribution between the age <65 years and ≥65 years groups (*p* = 0.944). As expected under the age-stratified design, the two age groups differed in their age distributions. Significant differences were observed in cognitive function between the two patient cohorts (*p* < 0.001), with age-stratified differences also evident in anxiety status (*p* < 0.001) and depressive status (*p* < 0.001). Among detected biomarkers, statistically significant differences were observed in all markers except Aβ1-42 (*p* = 0.351) between the two patient groups (*p* < 0.001).

We observed elevated concentrations of the axonal injury marker NfL in the ≥65 years group (*p* < 0.001). Similarly, GFAP—reflecting astrocytic reactivity—showed higher levels in the ≥65 years group (*p* < 0.001). Among phosphorylated tau proteins, both p-Tau217 and p-Tau181 concentrations were higher in the ≥65 years group than in the < 65 years group (*p* < 0.001). Regarding amyloid proteins, although no difference in Aβ1-42 was observed between the two groups, Aβ1-40 concentration was higher in the ≥65 years group (*p* < 0.001). Consequently, the Aβ42/40 ratio decreased in the ≥65 years group (*p* < 0.001).

### 3.2. Correlation Between Serum Biomarkers and Age

**Figure 2:**
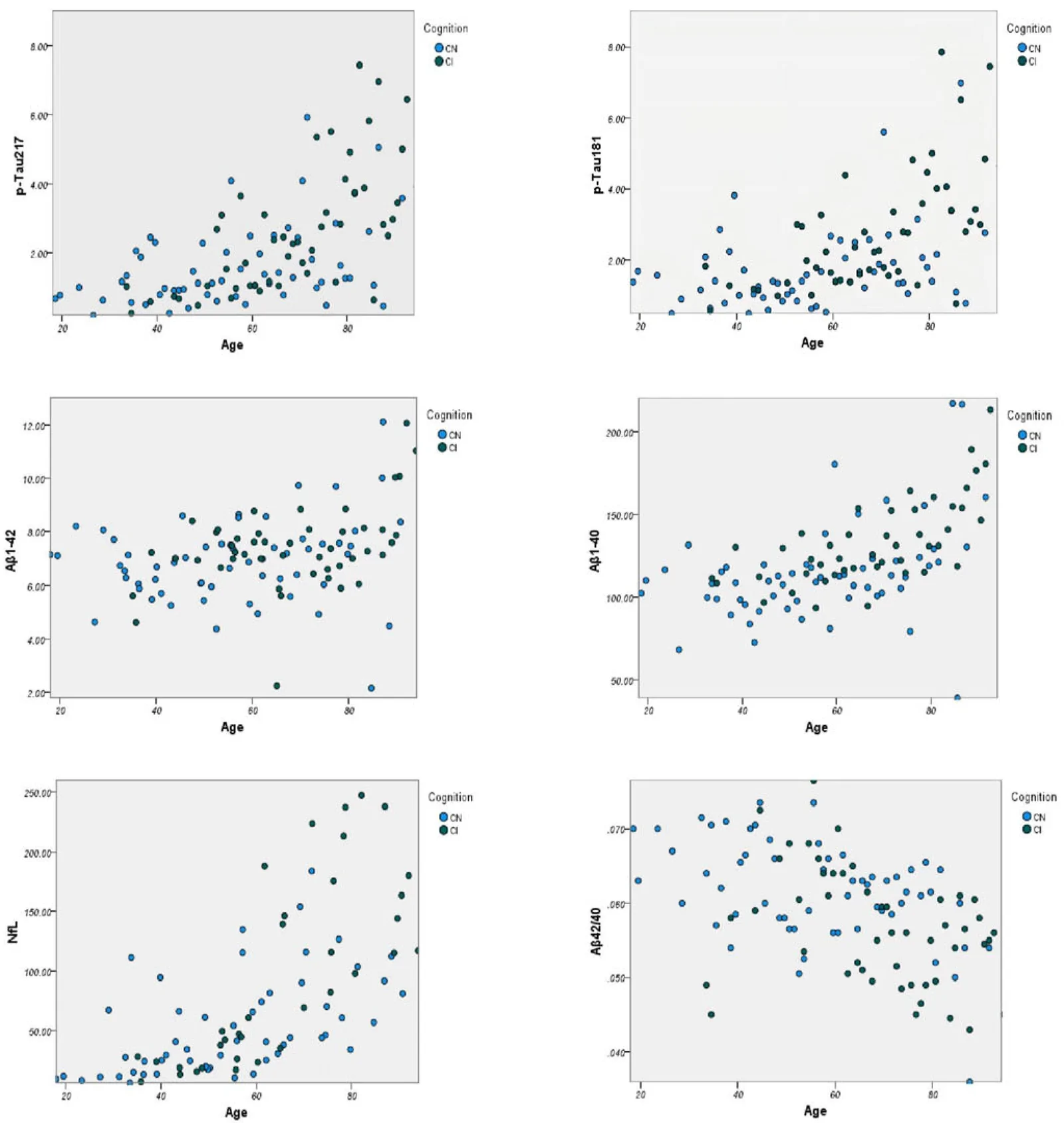
Scatter plots showing the associations between age and peripheral serum neurodegenerative biomarkers (Aβ1-40, Aβ1-42, NfL, p-Tau217, p-Tau181 [pg/mL for all protein markers], and Aβ42/40 [dimensionless]) in cognitively normal (CN, *n* = 238) and cognitively impaired (CI, *n* = 187) individuals. Associations were adjusted for sex and cognitive function. Abbreviations: Aβ, amyloid-beta; p-Tau, phosphorylated tau protein; GFAP, glial fibrillary acidic protein; NfL, neurofilament light chain; CN, cognitively normal; and MCI, mild cognitive impairment.

After adjusting for sex and cognitive function, the results demonstrated significant correlations between age and p-Tau217 (*r* = 0.275, *p* < 0.001), p-Tau181 (*r* = 0.288, *p* < 0.001), Aβ1-42 (*r* = 0.132, *p* = 0.007), Aβ1-40 (*r* = 0.254, *p* < 0.001), and NfL (*r* = 0.200, *p* < 0.001); all aforementioned indicators were positively correlated with age. Conversely, the ratio of Aβ1-42 to Aβ1-40 showed a significant negative correlation with age (*r* = −0.253, *p* < 0.001). It is noteworthy that the marker GFAP showed no significant correlation with age (*r* = 0.062, *p* = 0.207) (Table 2, Figure 2).

### 3.3. Differences in Biomarkers Across Cognitive Status Groups

**Figure 3:**
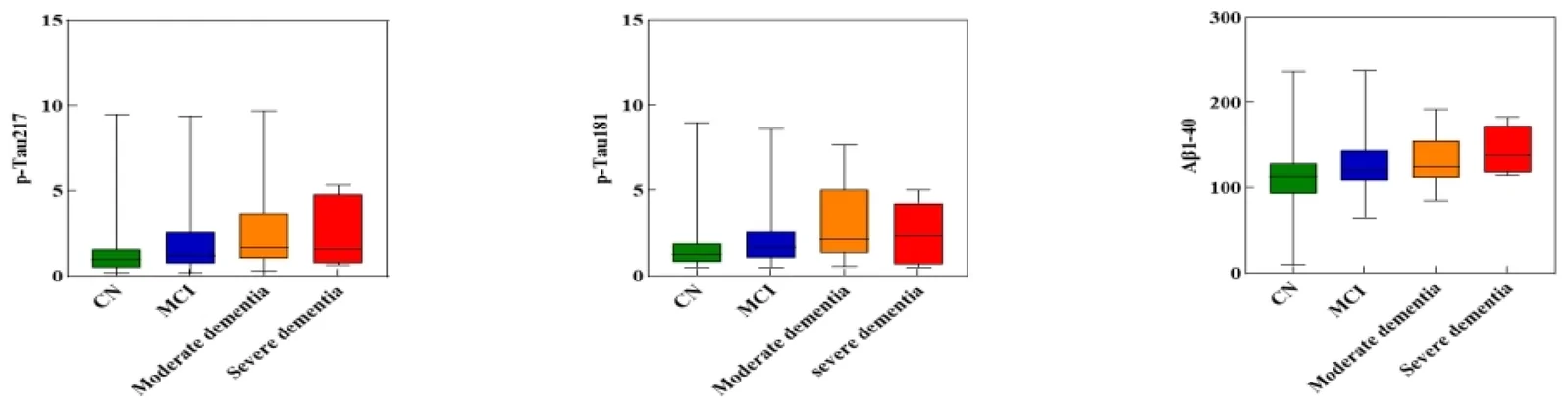

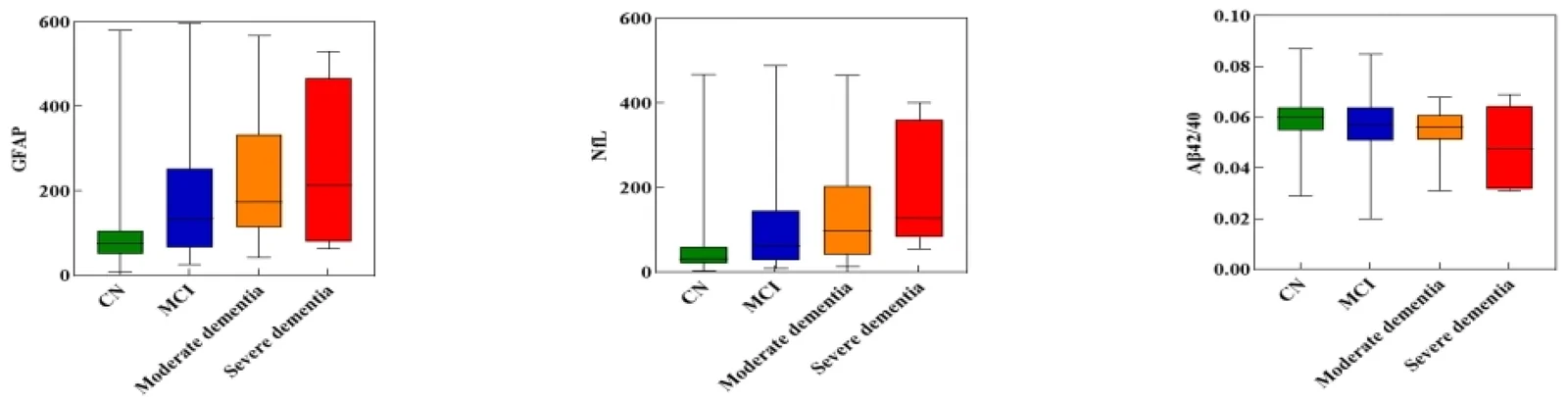
Box plots comparing serum biomarker levels (pg/mL for all markers except the Aβ42/40, which is a dimensionless ratio) across the cognitively normal (CN, *n* = 238), mild cognitive impairment (MCI, *n* = 141), moderate dementia (*n* = 40), and severe dementia (*n* = 6) groups. The upper and lower boundaries of each box represent the 75th and 25th percentiles, respectively; the horizontal line within each box indicates the median; whiskers extend to the minimum and maximum values within 1.5× the interquartile range. Abbreviations: Aβ, amyloid-beta; p-Tau, phosphorylated tau protein; GFAP, glial fibrillary acidic protein; NfL, neurofilament light chain; CN, cognitively normal; and MCI, mild cognitive impairment.

This study systematically compared serum-based neurodegeneration-associated biomarker levels across four groups in 425 samples: CN (*n* = 238), MCI (*n* = 141), Moderate dementia (*n* = 40), and Severe dementia (*n* = 6, considered exploratory due to small sample size) (Table 3, Figure 3). Results indicate that, apart from Aβ1-42 showing no significant distribution differences across groups (*χ*^2^ = 6.95, *p* = 0.073), all other biomarkers exhibited statistically significant intergroup variations (all *p* < 0.001), revealing progressive trends in biomarker levels with advancing cognitive impairment. Levels of p-Tau217 and p-Tau181, reflecting tau-associated neural injury, were significantly elevated in the Moderate dementia group compared to the MCI and CN groups. The Severe dementia group showed descriptively similar elevations, although statistical inference for this subgroup was precluded by its small size. The astrocytic reactivity marker GFAP showed significantly higher expression in the Moderate dementia group than in the MCI and CN groups, with MCI group levels also exceeding those of the CN group (*p* < 0.001). Although the Severe dementia group (*n* = 6) showed the numerically highest GFAP levels, this subgroup was too small for reliable statistical inference and was therefore considered exploratory. The neuroaxonal damage marker NfL similarly exhibited an increasing trend, with the Moderate dementia group showing significantly higher levels than the MCI and CN groups. The Severe dementia group followed a similar descriptive pattern, but the limited sample size precluded meaningful statistical comparison. Descriptively, Aβ1-40 levels were numerically highest in the Severe dementia group, and the Aβ42/40 ratio was numerically lowest in this group; however, given the small sample size, these descriptive findings should be interpreted with caution and are considered exploratory.

### 3.4. Analysis of Discrimination Performance Under Age Stratification

**Figure 4:**
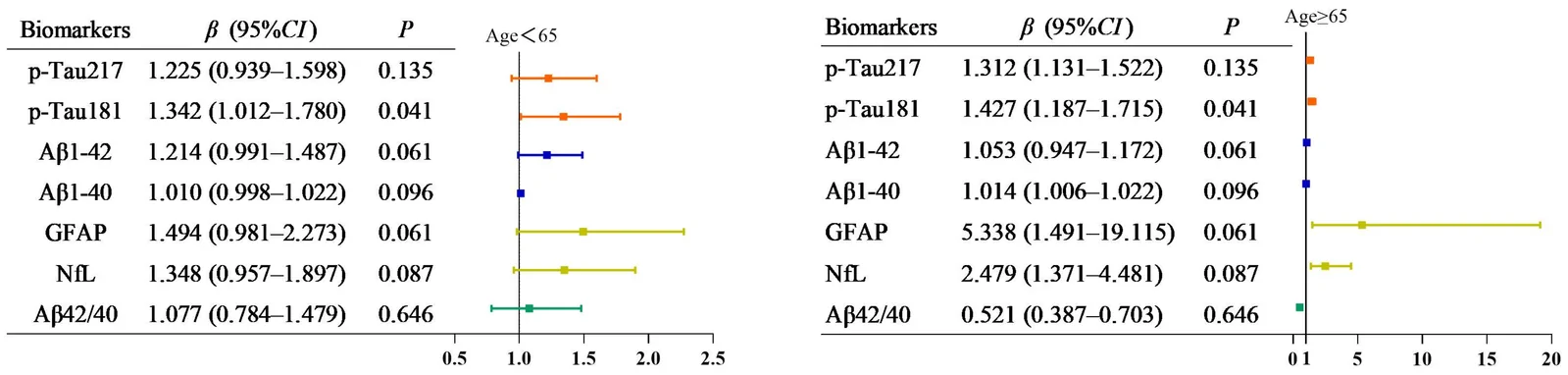
Forest plot of the associations between serum biomarkers and cognitive impairment (defined as MoCA score below the education-adjusted cutoff, encompassing MCI and moderate-to-severe dementia) across different age groups (<65 years, *n* = 182; ≥65 years, *n* = 243). Odds ratios are plotted on a logarithmic scale; the vertical dashed line indicates OR = 1.0. Abbreviations: Aβ, amyloid-beta; p-Tau, phosphorylated tau protein; GFAP, glial fibrillary acidic protein; NfL, neurofilament light chain; CN, cognitively normal; and MCI, mild cognitive impairment.

Within an age-stratified framework, we systematically evaluated the discriminative efficacy of circulating serum biomarkers for cognitive impairment. Logistic regression analyses were performed to calculate the odds ratios (OR) and 95% confidence intervals (95% *CIs*) for each biomarker associated with cognitive impairment in both the age <65 years and ≥65 years groups, with results visualized via forest plots (Table 4, Figure 4). In the ≥65 years group, p-Tau181 (*OR* = 1.427, 95% *CI*: 1.187–1.715, *p* < 0.001), p-Tau217 (*OR* = 1.312, 95% *CI*: 1.131–1.522, *p* < 0.001), GFAP (*OR* = 5.338, 95% *CI*: 1.491–19.115, *p* = 0.010), NfL (*OR* = 2.479, 95% *CI*: 1.371–4.481, *p* = 0.003) were significantly associated with higher odds of cognitive impairment, although the wide confidence interval for GFAP indicates limited precision of the estimate, likely attributable to the smaller subgroup size and the skewed distribution of GFAP values in this subgroup, whereas the Aβ42/40 ratio (*OR* = 0.521, 95% *CI*: 0.387–0.703, *p* < 0.001) showed a significant inverse association. In patients aged <65 years, only p-Tau181 (*OR* = 1.342, 95% *CI*: 1.012–1.780, *p* = 0.041) demonstrated independent discriminative capability after adjustment for sex, anxiety, and depression status.

**Figure 5:**
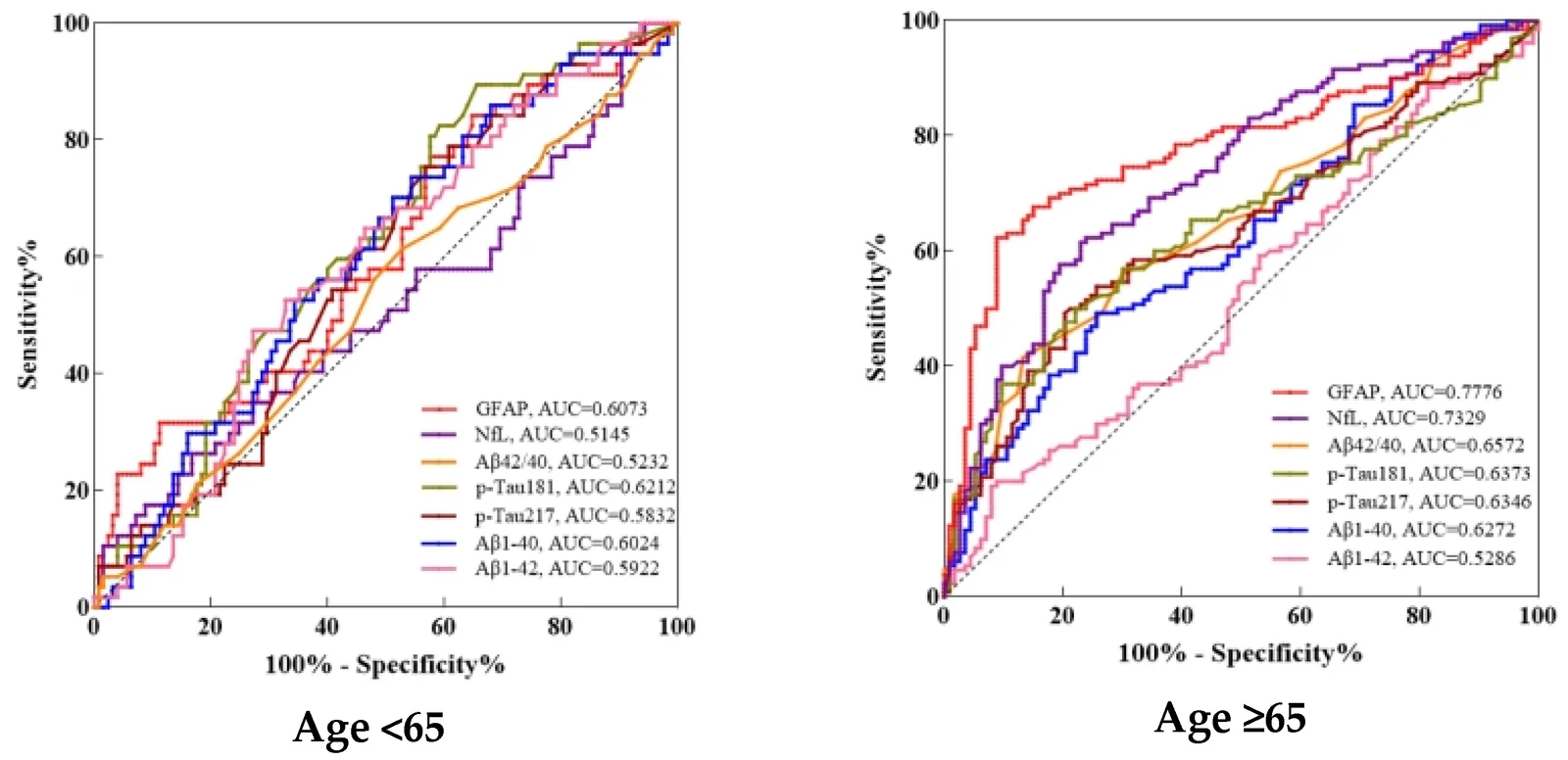
ROC curves for different serum biomarkers in predicting cognitive impairment stratified by age (<65 years, *n* = 182; ≥65 years, *n* = 243). The *x*-axis represents 1-specificity (%), and the *y*-axis represents sensitivity (%). Abbreviations: Aβ, amyloid-beta; p-Tau, phosphorylated tau protein; GFAP, glial fibrillary acidic protein; and NfL, neurofilament light chain.

We further evaluated the discriminative performance of each biomarker using ROC curve analysis (Figure 5, Table 5). In the ≥65 years group, GFAP showed the numerically highest AUC value (AUC = 0.7776), followed by NfL (AUC = 0.7329). However, formal statistical comparison of AUCs between biomarkers was not performed, so no biomarker can be declared statistically superior. In the <65 years group, p-Tau181 showed the numerically highest AUC value (AUC = 0.6212), although the modest AUC value suggests limited clinical utility as a standalone screening tool.

## 4. Discussion

In this exploratory cross-sectional analysis, we observed differences in the levels of circulating neurodegeneration-associated biomarkers and their associations with clinically defined cognitive impairment across age groups. Firstly, after controlling for confounding factors, age exhibited a negative correlation with Aβ42/40, reflecting amyloid metabolism imbalance, whilst showing positive correlations with p-Tau217, p-Tau181, and NfL reflecting tau-related neural injury and axonal injury, respectively. GFAP showed no significant correlation with age after adjusting for cognitive status, suggesting that its elevation may primarily reflect cognitive impairment burden rather than physiological aging. Secondly, most biomarkers (except Aβ1-42) exhibited significant gradient changes across the continuum from normal cognition to late-stage dementia, revealing their association with cognitive impairment severity. The lack of a significant difference in Aβ1-42 across cognitive status groups (*p* = 0.073) may be due to its relatively low serum concentrations and greater analytical variability, its potential plateau in advanced dementia, and the limited sample size of moderate-to-severe dementia. Most critically, age-stratified analysis revealed a differential association pattern: among patients aged <65 years, only p-Tau181 demonstrated independent discriminatory value; whereas in patients aged ≥65 years, multiple biomarkers were significantly correlated with cognitive impairment. However, this age-related difference may be partly driven by the higher proportion of moderate-to-severe dementia in the older group, which could inflate the AUC values for GFAP and NfL in that group. These findings should be considered exploratory, as formal interaction testing was not performed. This age-related association pattern can be explained by age-related physiological changes in the nervous system and the progression of brain pathological damage. These biomarkers are closely linked to Alzheimer’s disease-related pathological changes, including β-amyloid deposition and hyperphosphorylated tau protein [41,42]. The present study did not conduct amyloid/tau PET or CSF testing to confirm definite Alzheimer’s disease pathology. p-Tau181, as a well-recognized indicator of tau-associated neural damage, may show abnormalities at relatively early stages of cognitive decline. Therefore, in younger inpatient populations, cognitive impairment is more likely to be accompanied by elevated p-Tau181 levels, making this marker a valuable indicator for cognitive assessment in this population. In contrast, after entering the elderly stage, increased neuroinflammatory responses, altered blood–brain barrier function, and cumulative brain damage jointly lead to more extensive neurodegeneration [27]. At this point, the levels of GFAP reflecting astrocytic reactivity and NfL reflecting neuronal damage rose significantly [43,44]. These changes are not limited to Alzheimer’s disease pathology, but also result from combined effects of neuroinflammation and age-related degenerative lesions [45,46]. Therefore, for elderly inpatients, GFAP and NfL may reflect different aspects of neurodegeneration, and both showed numerically higher AUC values in the elderly group in our descriptive analysis. These findings suggest that different pathophysiological processes may be involved in different age groups. However, the cross-sectional design precludes any inference about temporal ordering.

Previous studies have proposed a hypothetical temporal model in which amyloid-β dysregulation is an early initiating event [16,17,47], followed by the aggregation of hyperphosphorylated tau (p-Tau181 and p-Tau217) as a key driver of neuronal dysfunction [18], and later by glial activation (GFAP) and neuronal damage (NfL) as the disease progresses [48]. This dynamic biomarker model has been supported by longitudinal cohort studies [49,50]. Our cross-sectional data cannot confirm this temporal sequence, but provide exploratory evidence that different biomarkers may show different patterns across age groups, warranting further investigation in longitudinal cohorts. This observed pattern is consistent with the possibility that axonal degeneration and amyloid pathology may jointly contribute to cognitive decline beyond cortical atrophy alone [51]. However, these findings should be interpreted as cross-sectional associations rather than evidence of temporal progression. Our age-stratified analysis showed that p-Tau181 had relatively better discriminative performance in the younger inpatient group (<65 years), while GFAP and NfL, reflecting more advanced or cumulative damage, showed numerically higher AUC values in the elderly (≥65 years). This raises the question of what mechanisms drive the age-related peripheral manifestation of these markers, prompting us to consider the role of blood–brain barrier integrity.

The role of blood–brain barrier (BBB) integrity cannot be overlooked when interpreting peripheral serum biomarkers [52]. Physiological aging is associated with structural and functional alterations of the BBB, including pericyte loss and tight junction disruption [53]. These changes not only facilitate the leakage of brain-derived proteins, such as p-Tau, NfL, and GFAP, into the peripheral circulation [54,55] but may also exacerbate neuroinflammation by promoting the infiltration of peripheral immune cells [52]. Therefore, elevated blood levels of these biomarkers in elderly individuals may partly reflect age-related BBB breakdown rather than Alzheimer’s disease-specific brain pathology alone [56,57]. Our finding that NfL and GFAP were particularly elevated in the ≥65 years group further supports that the peripheral manifestation of these markers is heavily influenced by the cumulative effects of age on both neurodegeneration and vascular-immune barriers.

Our findings both align with and introduce novel insights into previous high-quality studies, most of which have focused on plasma neurodegenerative biomarkers. However, it is important to note that our study used serum samples, whereas many previous studies used plasma. Serum and plasma differ in their protein composition, clotting factor content, and matrix effects, which may affect absolute biomarker concentrations and assay performance. Therefore, direct comparison of absolute concentrations and diagnostic performance between our study and plasma-based studies should be interpreted with caution. Nevertheless, the relative patterns and associations observed in our study are broadly consistent with those reported in plasma-based cohorts. Multiple prior studies [17,18,19,48,51,58] have confirmed the association between serum biomarkers such as p-Tau217, p-Tau181, and NfL with age and cognitive decline, aligning with the overall correlation trends observed in our study. However, we found a weaker overall correlation between GFAP and age, yet significant differences across distinct cognitive status groups, with the strongest association observed in the elderly group with cognitive impairment. This contrasts with findings from some studies reporting a significant positive correlation between GFAP and age, suggesting that the clinical significance of GFAP may be highly dependent on specific age groups, disease stages and clinical settings [59,60]. Its application value appears more prominent in elderly individuals and patients with cognitive impairment accompanied by marked astrocytic reactivity. Therefore, when interpreting astrocytic reactivity markers such as GFAP, stratified analysis considering specific clinical contexts is essential. The innovation of this study lies in exploring the age-stratified application value of peripheral serum neurodegeneration biomarkers among neurological inpatients with complex comorbidities. At present, research focusing on age-stratified discriminative performance of these biomarkers in hospitalized populations is still limited. This stratified analysis framework helps to realize more personalised and precise clinical assessment for inpatients with cognitive concerns, and has potential clinical translational value.

Naturally, our study also presents several inevitable limitations, which should be fully acknowledged. A particularly important limitation is the spectrum bias introduced by the unequal distribution of cognitive severity between age groups. The ≥65 years group contained a higher proportion of moderate-to-severe dementia cases. This spectrum bias may have inflated the AUC values for GFAP and NfL in this group and may partly account for the observed age-related differences. Therefore, we cannot exclude the possibility that these differences are partly driven by differences in disease severity rather than by age-specific biology. Future studies with better-matched cognitive severity across age groups are warranted. Several other limitations should also be acknowledged. Firstly, the cross-sectional design precludes inferring causal relationships between biomarkers and cognitive impairment, nor can it depict the dynamic trajectories of individual biomarkers across age and disease progression. Secondly, all participants were neurological inpatients with various comorbidities, which may interfere with the detection results of serum biomarkers; although we have adjusted for major confounding factors, potential residual confounding cannot be completely ruled out. Thirdly, subjects were grouped purely by clinical neuropsychological scales, without using amyloid/tau PET or CSF biomarkers to confirm Alzheimer’s disease pathology. Thus, we cannot distinguish cognitive impairment caused by Alzheimer’s disease from that induced by other neurodegenerative diseases. Fourthly, this is a single-centre study based on neurological inpatients, so the research findings cannot be directly generalised to community-dwelling populations or asymptomatic individuals. Moving forward, we shall conduct prospective longitudinal cohort studies and multi-centre validation to further verify the discriminative value and dynamic changing rules of these age-stratified biomarkers. Additionally, although we excluded individuals with clinically diagnosed psychiatric disorders, some participants exhibited moderate-to-severe anxiety or depressive symptoms as reflected by scale scores. While we adjusted for anxiety and depression scores in the regression models, we cannot completely rule out residual confounding from educational level or specific primary neurological diagnoses, or the potential impact of concomitant medications that were not systematically recorded. Another limitation is that we did not systematically record the specific primary neurological diagnoses (e.g., cerebrovascular disease, Parkinsonian disorders, epilepsy, neuropathy) within our inpatient cohort. As a result, we cannot exclude the possibility that certain underlying conditions may have influenced serum biomarker levels or cognitive performance independently of age. However, our objective was to evaluate the general discriminative performance of these biomarkers for cognitive impairment across a real-world neurology inpatient population, rather than to differentiate between specific etiologies. The heterogeneity of the inpatient population reflects the actual clinical scenario in which these biomarkers would be applied. Future studies with detailed diagnostic subclassification are warranted to confirm the generalizability of our findings. In addition, the severe dementia group comprised only 6 participants, which precludes reliable statistical inference for this subgroup. While we included these cases in the descriptive comparisons to reflect the full spectrum of cognitive impairment, we acknowledge that the findings specific to the severe dementia stage should be interpreted with caution and are exploratory in nature. The limited sample size also prevented meaningful subgroup-specific analyses for this stage. Additionally, an analysis comparing CN and MCI groups was not performed in this study. Future research should investigate whether these serum biomarkers can differentiate early cognitive impairment (CN vs. MCI), as this may have important implications for early screening. We also acknowledge that data on certain potential confounders, including hypertension, diabetes mellitus, history of cerebrovascular disease, and renal function (eGFR), were not systematically collected in this study. Consequently, we were unable to adjust for these factors in our regression models. Although this limits our ability to exclude confounding from vascular comorbidities completely, our primary objective was to evaluate the discriminative performance of serum biomarkers for cognitive impairment in a real-world inpatient setting. Future studies with more comprehensive covariate collection are warranted to confirm the robustness of our findings. Additionally, although we performed age-stratified analyses to explore age-related patterns, we did not formally test age-by-biomarker interaction terms in a multivariable model. Therefore, the observed differences in odds ratios between age groups should be interpreted as exploratory patterns rather than definitive evidence of effect modification. Furthermore, we did not formally compare AUCs between age groups using DeLong’s test. Although GFAP and NfL showed numerically higher AUC values in the older group, this descriptive difference was not statistically tested. Therefore, we cannot conclude that biomarker performance is superior in one age group over the other. Future studies with larger sample sizes and formal interaction testing are warranted to validate these exploratory findings. What’s more, our findings are based on serum samples; the results may not be directly interchangeable with studies using plasma, and future studies should directly compare serum and plasma measurements to establish matrix-specific reference values. In addition, independent analytical validation specifically for serum was not performed in our laboratory, which should be considered when interpreting the absolute concentrations reported here.

## 5. Conclusions

In this exploratory cross-sectional study of neurological inpatients, we observed that the association strength and discriminative performance of serum neurodegeneration-associated biomarkers for clinically defined cognitive impairment may differ across age groups. p-Tau181 showed relatively better discriminative performance in patients younger than 65 years, while GFAP and NfL showed numerically higher AUC values in those aged 65 years and above. However, the overall discriminative ability of individual biomarkers was modest (AUC < 0.70 in the younger group), and these findings should be considered exploratory rather than definitive. They do not support the use of any single serum biomarker as a standalone screening tool for cognitive impairment. Instead, our results suggest that age-stratified interpretation may be valuable, and that comprehensive clinical evaluation remains essential. Future longitudinal, multi-centre studies with formal interaction testing and validation in independent cohorts are warranted to confirm these exploratory observations and to clarify their potential clinical utility.

## Figures and Tables

**Table 1:** Comparison of baseline data among patients of different age groups.

Variables	Age < 65 Years (*n* = 182)	Age ≥ 65 Years (*n* = 243)	*p*
**Age**	55 (45, 61)	73 (69, 79)	-
**Sex**			
Male	87 (47.80)	117 (48.15)	0.944
Female	95 (52.20)	126 (51.85)	
**Cognition**			
CN	125 (68.68)	113 (46.50)	<0.001
MCI	50 (27.47)	91 (37.45)	
Moderate dementia	7 (3.85)	33 (13.58)	
Severe dementia	-	6 (2.47)	
**Anxiety stage**			
Normal	127 (69.78)	110 (45.27)	<0.001
Mild	40 (21.98)	89 (36.62)	
Moderate	15 (8.24)	37 (15.23)	
Severe	-	7 (2.88)	
**Depression State**			
Normal	152 (83.52)	131 (53.91)	<0.001
Mild	29 (15.93)	90 (37.04)	
Moderate	-	14 (5.76)	
Severe	1 (0.55)	8 (3.29)	
**Biomarkers**			
p-Tau217	0.82 (0.51, 1.40)	1.37 (0.80, 2.70)	<0.001
p-Tau181	1.24 (0.73, 1.67)	1.77 (1.16, 2.71)	<0.001
Aβ1-42	7.15 (5.95, 8.17)	7.11 (5.87, 8.65)	0.351
Aβ1-40	113.18 (92.21, 126.07)	124.65 (107.68, 148.92)	<0.001
GFAP	63.55 (45.22, 86.55)	118.17 (83.37, 224.10)	<0.001
NfL	27.16 (17.03, 46.87)	61.53 (35.33, 139.16)	<0.001
Aβ42/40	0.07 (0.06, 0.07)	0.06 (0.05, 0.06)	<0.001

Note: Data are presented as median (Q1, Q3), where Q1 and Q3 denote the 25th and 75th percentiles, respectively; categorical data are presented as n (%). Between-group comparisons for continuous variables were performed using the Mann–Whitney U test, and categorical variables were compared using the chi-square test. Age is the grouping variable; therefore, the *p*-value for age is not applicable. The age range for the <65 years group was 18–64 years, and for the ≥65 years group was 65–94 years. Abbreviations: Aβ, amyloid-beta; p-Tau, phosphorylated tau protein; GFAP, glial fibrillary acidic protein; NfL, neurofilament light chain; CN, cognitively normal; and MCI, mild cognitive impairment.

**Table 2:** Correlation between age and peripheral serum neurodegenerative biomarkers (adjusted for sex and cognitive status).

Variables	*r*	*p*
p-Tau217	0.275	<0.001
p-Tau181	0.288	<0.001
Aβ1-42	0.132	0.007
Aβ1-40	0.254	<0.001
GFAP	0.062	0.207
NfL	0.200	<0.001
Aβ42/40	−0.253	<0.001

Note: Partial Spearman’s correlation analysis was used for statistical calculation. *p*-values were adjusted for multiple comparisons using the Benjamini–Hochberg false discovery rate (FDR) method. Adjusted *p* < 0.05 was considered statistically significant. Abbreviations: see Table 1.

**Table 3:** Comparison of peripheral serum neurodegenerative biomarkers across different cognitive status groups.

Variables	Total (*n* = 425)	CN (*n* = 238)	MCI (*n* = 141)	Moderate Dementia (*n* = 40)	Severe Dementia * (*n* = 6)	*p*
Aβ42/40	0.06 (0.05, 0.07)	0.06 (0.06, 0.07) ^a^	0.06 (0.05, 0.07) ^a^	0.06 (0.05, 0.07) ^ab^	0.05 (0.03, 0.06) ^b^	<0.001
p-Tau217	1.09 (0.65, 2.06)	0.98 (0.51, 1.58) ^b^	1.23 (0.77, 2.57) ^ab^	1.67 (1.11, 3.67) ^a^	1.60 (0.85, 4.01) ^ab^	<0.001
p-Tau181	1.47 (0.93, 2.31)	1.29 (0.82, 1.89) ^b^	1.70 (1.08, 2.58) ^ab^	2.16 (1.38, 4.79) ^a^	2.33 (0.90, 3.82) ^ab^	<0.001
Aβ1-42	7.12 (5.92, 8.45)	6.90 (5.80, 8.34) ^a^	7.21 (5.97, 8.41) ^a^	7.35 (6.75, 9.95) ^a^	7.27 (5.91, 9.19) ^a^	0.073
Aβ1-40	118.32 (101.53, 136.64)	114.16 (93.20, 129.23) ^b^	120.74 (108.09, 144.17) ^ab^	125.02 (112.97, 154.16) ^ab^	138.94 (122.58, 164.29) ^a^	<0.001
GFAP	89.35 (61.46, 162.66)	76.09 (51.31, 106.67) ^c^	137.22 (67.01, 281.64) ^b^	173.41 (116.78, 321.14) ^ab^	213.85 (106.88, 399.75) ^a^	<0.001
NfL	43.64 (24.57, 96.78)	31.86 (21.81, 59.88) ^c^	61.63 (29.79, 142.99) ^b^	98.69 (41.81, 186.95) ^ab^	127.59 (95.90, 299.70) ^a^	<0.001

Note: Data are presented as median (Q1, Q3), where Q1 and Q3 denote the 25th and 75th percentiles, respectively. The Kruskal–Wallis H test was used for inter-group comparisons. *p*-values were adjusted for multiple comparisons using the Benjamini–Hochberg false discovery rate (FDR) method. Post-hoc pairwise comparisons were performed using the Dunn test with Bonferroni correction. Within each row, values with different superscript letters (a, b, c) indicate statistically significant differences between groups (adjusted *p* < 0.05); values sharing the same superscript letter indicate no statistically significant difference. * The severe dementia group contains only 6 participants. This subgroup was included in the statistical comparisons for descriptive completeness; however, results for this subgroup should be interpreted with caution and are considered exploratory. Abbreviations: see Table 1.

**Table 4:** Logistic regression analysis of the association between serum biomarkers and cognitive impairment.

Variables	Age < 65 Years		Age ≥ 65 Years	
	*OR* (95% *CI*)	*p*	*OR* (95% *CI*)	*p*
p-Tau217	1.225 (0.939–1.598)	0.135	1.312 (1.131–1.522)	<0.001
p-Tau181	1.342 (1.012–1.780)	0.041	1.427 (1.187–1.715)	<0.001
Aβ1-42	1.214 (0.991–1.487)	0.061	1.053 (0.947–1.172)	0.338
Aβ1-40	1.010 (0.998–1.022)	0.096	1.014 (1.006–1.022)	<0.001
GFAP	1.494 (0.981–2.273)	0.061	5.338 (1.491–19.115)	0.010
NfL	1.348 (0.957–1.897)	0.087	2.479 (1.371–4.481)	0.003
Aβ42/40	1.077 (0.784–1.479)	0.646	0.521 (0.387–0.703)	<0.001

Note: OR, odds ratio; CI, confidence interval. The dependent variable was cognitive impairment status (impaired vs. normal). Each biomarker was evaluated in a separate logistic regression model. All models were adjusted for sex, anxiety status, and depression status. ORs correspond to a 1-unit increase in each biomarker (pg/mL for all markers except Aβ42/40, which is per 0.01-unit increase). Abbreviations are as defined in Table 1.

**Table 5:** ROC-derived performance metrics of serum biomarkers for cognitive impairment across age groups.

Biomarkers	Age < 65 Years				Age ≥ 65 Years			
	AUC (95% *CI*)	Cutoff	Sensitivity	Specificity	AUC (95% *CI*)	Cutoff	Sensitivity	Specificity
p-Tau217	0.5832 (0.497–0.669)	0.705	0.754	0.432	0.6346 (0.565–0.704)	1.860	0.492	0.796
p-Tau181	0.6212 (0.537–0.705)	0.805	0.895	0.344	0.6373 (0.567–0.707)	2.120	0.500	0.779
Aβ1-42	0.5922 (0.506–0.678)	7.635	0.474	0.728	0.5286 (0.456–0.601)	9.795	0.192	0.920
Aβ1-40	0.6024 (0.516–0.689)	104.585	0.737	0.456	0.6272 (0.558–0.697)	131.905	0.492	0.743
GFAP	0.6073 (0.518–0.696)	117.475	0.316	0.888	0.7776 (0.718–0.837)	150.280	0.623	0.912
NfL	0.5145 (0.420–0.609)	63.720	0.263	0.832	0.7329 (0.670–0.796)	74.410	0.615	0.770
Aβ42/40	0.5232 (0.432–0.615)	0.0635	0.614	0.472	0.6572 (0.589–0.725)	0.0535	0.585	0.133

Note: AUC, area under the curve; *CI*, confidence interval. Cutoff values were determined by maximizing the Youden index from ROC curve analysis. Sensitivity and specificity are reported at the corresponding optimal cutoff. Abbreviations are as defined in Table 1.

## Data Availability

The data presented in this study are available upon request from the corresponding author due to privacy and ethical restrictions. The data are not publicly available as they contain information that could compromise the privacy of research participants, and the informed consent obtained from participants did not include provisions for public data sharing.
